# Abstracts from Foreign Journals

**Published:** 1901-08

**Authors:** M. P. Ravenel, S. J. J. Harger

**Affiliations:** Philadelphia, Pa.; Philadelphia, Pa.


### ABSTRACTS FROM FOREIGN JOURNALS.

### FRENCH.

UNDER THE DIRECTION OF M. P. RAVENEL, M.D., AND S. J. J. HARGER, V.M.D., OF PHILADELPHIA, PA.

SERO-DIAGNOSIS OF TUBERCULOSIS. In a series of communications to the Academy of Sciences, 1898, Arloing demonstrated that the sero-agglutination constitutes a procedure of great value in the early diagnosis of tuberculosis in man.

While experimenting upon the human species Arloing also made analogous observations upon bovines, and found that, as a general rule, the blood serum of adult bovidæ agglutinates the bacillus of Koch in a homogeneous culture. This agglutinating power is feeble, while that of the blood serum of tuberculosis bovidæ is much greater. The difference is sufficiently marked to enable one to say whether the serum is obtained from a healthy or a tuberculous subject.

The technique of the operation is little complicated. Two elements are necessary: 1. A homogeneous culture of the tubercle bacillus. 2. Blood serum of the subject in question. The cultures and the serum are then placed in a number of glass tubes and mixed by shaking the tube. The agglutination of the bacilli commences in five or six hours, and ceases in twenty-four hours. It is characterized by a flocculent appearance of the mixture in the tube. The flocculi increase, and are deposited in the bottom of the tube, leaving the upper strata of the liquid perfectly transparent when agglutination is completed. When the culture and serum are mixed in the proportion of four drops of culture and one drop of serum, the latter is said to have an agglutinating power of one-fifth.

The experiments of Arloing have demonstrated: 1. That the blood serum of calves has no agglutinating power, not even one-half. 2. That the agglutinating power of the serum of a healthy non-tuberculous ox is about one-fifth. 3. That in the tuberculous animal this power is one-tenth and higher. A suspected bovine in which the serum shows this degree of agglutination can be considered tuberculous. The clinical application of this test may be made with advantage in animals which have been previously inoculated with tuberculin, and, therefore, will not react to this agent at the time. This diagnosis can only be made accurately in a bacteriological laboratory.—*Rec. de Méd. Vét.*

Kerophocele Caused by Forging. Menveux observed a kerophocele in the right hind foot. It was as large as a walnut, caused absorption of the anterior face of the third phalanx, dropping of the sole, and suppuration of the podophylous tissue. Extirpation of the horn tumor was necessary. The foot had been shod with a shoe truncated at the toe. The toe of the wall was not rasped off; the forging still continued, but the sound was not so apparent.—*Rec. de Méd. Vét.*

Ulceration of the Pituitary Mucous Membrane Simulating Glanders. The mare which had shown these symptoms for three years had come under Nocard's observation. During this period she had been malleinized twice at intervals of a month by Müller, but gave no reaction. Condemned for glanders, and mallein considered a failure.

*Symptoms.* The animal presented every indication of good health. A slight yellowish bilateral nasal discharge, sometimes bloodstained, dry, and scabby on its surface, and adhering to the skin of both nostrils. Submaxillary glands not enlarged, excepting a few small hypertrophied ganglia, insensitive, and movable under the skin. The mucous membrane on both sides of the septum nasi and on the ends of the turbinated bones as far up as the eye and the finger could detect was thickened and covered with yellowish-red, feebly adherent scabs. Under the latter was an irregular, granular, reddish, hemorrhagic surface, whose aspect was quite different from the characteristic lesions of chronic glanders. Instead of a necrotic destruction of tissue, punched-out ulcerations, with salient indurated borders, there were irregularly outlined thickenings of the mucous membrane whose most salient part was ulcerated. Acute glanders sometimes shows analogous lesions, but their evolution passes very rapidly, and is accompanied by a grave general state of the organism. A third injection of mallein was made with negative results. Inoculations of guinea-pigs also were negative. Glanders and tuberculosis were eliminated. The symptoms remained unchanged.

*Necropsy.* The aforesaid lesions of the inferior part of the nasal septum and turbinated bones, and superior part of the septum were normal.

*Larynx and Trachea.* Two small oval ulcerations on the posterior face of the epiglottis, clearly outlined and with salient indurated borders, resembling chronic glanderous chancres; confluent ulcerations at the base of the epiglottis; ulcerative destruction of the mucous membrane of the right ventricle; ulceration on the anterior face below the cricoid cartilage; five or six elongated ulcerations in the upper half of the trachea. *Bronchi and lungs* normal.

En résumé, the lesions were confined to the upper respiratory tract. Those in the nose were readily differentiated from the alterations of chronic glanders, retained the same aspect for several years, did not cause induration of the submaxillary glands, and had nothing in common with glanders. On the contrary, the lesions in the larynx and trachea appeared identical with those of chronic glanders. Had the history of the case been unknown, the reporter, basing his opinion entirely upon the necropsy, would have pronounced the case glanders. Histological examination gave no better information.

The disease was not considered to be glanders, but its identity could not be determined.—*Rec. de Méd. Vét.*

Normal Temperature of the Mule. The animal temperature varies with the species, the individuals of the same species, and the conditions of the exterior. Pader found the temperature in 500 mules to oscillate from 37.3° to 39° C., the minimum night atmosphere temperature being 22°, and the maximum during the day 30°. Temperatures above 38.7° were eliminated as presenting some abnormal condition. The average temperature of the entire number was 38.0226°. The largest number—62 and 63—showed a temperature of 37.8° and 37.9° respectively, which may be considered as the normal average.—*Rec. de Méd. Vét.*

Osteoma of the Aponeuroses of the Thigh. The horse presented on the external face of the femoral region a flat, hard tumor, sharply delimitated and developed within the superficial aponeurosis covering the muscles of this region. It was successfully removed. There was no lameness.

The tumor consisted of a flat plate, 25 cm. long, 10 cm. wide, and 5 to 10 mm. thick, which, on histological examination, was found to consist of true bone osteoma.

[Dr. John W. Adams has shown to the writer a similar plate of bone taken from the postero-external aspect of the thigh, where it had existed for some years until the death of the animal.—S. J. J. H.]—*Rec. de Méd. Vét.*

Diabetic Urine from Complete Atrophy of the Pancreas. The animal, a fox-terrier bitch, presented the usual symptoms of diabetes: marked and progressive emaciation, ravenous appetite, excessive thirst and polyuria; Fehling's solution revealed sugar in the urine.

*Diet.* Mush made from bread, meat, and legumens. The food passed undigested.

*Treatment.* Twenty-five cgm. of antipyrine daily subcutaneously. No amelioration.

*Necropsy.* No marked alteration except those of the pancreas. The region corresponding to the gland was pigmented, and the gland itself was indicated only by some white, hard granulations.

*Microscopical Examination.* These granulations consisted of fibrous tissue, in the interior of which were found disseminated masses of small, transparent epithelial cells quite different from the normal pancreatic cells from which they are derived, and recall somewhat the arrangement of the cell masses in encephaloid cancer. Their nuclei stained readily with hæmatin and carmine. The arterioles are the seat of periarteritis and endarteritis, and, in some cases, of calcification. Vestiges of the gland and microscopical nerve ganglia were also found. This bitch had previously given birth to three puppies. There was a white discharge from the vulva, and from this time on she emaciated very rapidly.

The atrophy of the pancreas seems to have followed parturition. Was it caused by puerperal infection? The animal presented the characteristics of pancreatic or emaciating diabetes: rapid course, intestinal troubles, and rapid emaciation.—*Rec. de Méd. Vét.*

Atrophy of the Semimembranous Muscle following Castration. Monserrat castrated the animal and docked the tail. The animal, which was down twenty-five minutes, strongly resisted, pulling the leg toward the shoulder, the thigh muscles being rigidly contracted. About two months after the atrophy of the semimembranous was marked. The treatment consisted of stimulating applications, electricity, and exercise. The muscle regenerated itself.

The reporter attributes the lesion to compression of the ischo-tibial nerves by the rigidly contracted thigh muscles.—*Rec. de Méd. Vét.*

Destruction of Vermin on Chickens. An easy method of destroying vermin on chickens consists in allowing them to puddle for some days in a mixture of the following proportions placed in an excavation in the ground and under cover: One bucketful of coal or wood ashes, one pint of air-slaked lime, and a handful of sulphur. In ten to fifteen days the legs and toes scale off, and the parasite has disappeared.—*Rec. de Méd. Vét.*

Protargol in Purulent Arthritis. Protargol is an albuminate of silver, very staple, and contains 8.3 per cent. of the metal. It exists in the form of a yellowish powder soluble in water, glycerin, and blood serum. It possesses the bactericidal properties of nitrate of silver without being caustic or even irritant to the most delicate mucous membrane, and can be used for antiseptic purposes.

In man it is employed against specific affections of the urethra. Hendrickx obtained remarkable results in two instances in the horse. The first case was one of purulent traumatic arthritis of the hock, so grave in spite of all treatment that the animal was about to be destroyed. Thirty grammes of a 3 per cent. solution of protargol were injected into the joint. The animal improved at once, and rapidly recovered. The second case was a suppurated wound of the external lateral cartilage of the foot, with separation of the heel in a horse in which six months before both plantar nerves had been resected above the fetlock. After removing the quarter and daily applying a dressing of a 3 per cent. solution of protargol the animal recovered rapidly. This case shows that suppuration in a neurectomized foot is not necessarily fatal. In other hands the results with this drug have not been so happy, and its real value requires further demonstration.—*Rec. de Méd. Vét.*

Neurectomy in Surgical Therapeutics. Jacoulet asserts that low plantar neurectomy is insufficient for the purpose intended, because the posterior digital branch does not always exclusively supply the diseased area. Cases usually designated *navicular disease* may consist not only of a podo-sessamoidal synovitis with osteoarthritic lesions of the phalangeal articulation, but may be accompanied by a more or less marked lesion of osteoarthritis of the coronary joint as well as other synovial, ligamentous, and bony lesions situated below the coronet. Low neurectomy also leaves a cicatrix, and is often complicated with neuromas. The good effects of the low operation continue from fifteen months to two years. However, an operation whose good effects are of such duration cannot be entirely cast to one side. High plantar neurectomy has two disadvantages: degenerative processes in the foot and its effectiveness against lesions situated only below the fetlock. The low operation may be done on one side, and the high on the other, where the lesion is more extensive. Hence, during the last four years the author has substituted resection of the median and of the sciatic nerves. These nerves supply the regions where most of the causes of lameness are located—below the knee and the hock.

The operation is not advised when the lesions are accompanied by acute symptoms. The animal should have forty days' rest, not "turned out," and only put to work gradually. It is also asserted that the operation lessens the growth of the lesion as compared with the course of a similar lesion not neurectomized.

Since 1894 Jacoulet performed fifty-two median and three sciatic neurectomies. Of the latter one was for a spavin, and without any result; the other for large ring-bones, and with good results. Among the former were driving horses, hunters, and steeple-chasers. No degenerative processes were seen, excepting a breaking down of the tendons in one case in which the low and high plantar and the median operations were performed successively during a period of three years. Sometimes an eczematous condition of the coronet, which yielded to dissecting applications, was noticed.

The author concludes: mesoneurectomy is much superior to the high and low plantar resection ; it covers a larger area, and preserves the foot from degenerative processes, the ulnar nerve contributing to form the external plantar. Practised early it arrests the growth of the lesions. It requires only one operation, and leaves no visible blemish.

Resection of the sciatica, which, exclusively forming both plantars, suppresses all nerve influence in the inferior part of the member, requires a little more reserve.—*Rec. de Méd. Vét.*

Antiseptic Soaps. The virtues of antiseptic soaps are often highly extolled to the practitioner. Coremans experimented with soaps containing corrosive sublimate and formalin in solutions very diluted as well as of a strength ordinarily used in practice. He concludes : Antiseptic soaps have no or only a feeble action ; they give a false guarantee, and their price is not in accord with their virtue. Serafini considers them less efficacious than ordinary soaps in consequence of their diminished solubility.—*Annales de Méd. Vét.*

ANEURISM OF THE INFERIOR CERVICAL ARTERY. Aneurisms in the horse are very rare. Mouquet cites a case seen in a horse examined for a suspected lameness. Over the left sterno-humeralis muscle was a swelling half as large as an unshelled cocoanut, elastic, soft, diminishing on pressure, and imparting to the fingers a vibratory sensation comparable to that produced by the vibration of the wings of a fly held in the fingers close to a window pane. Auscultation revealed a continuous vibratory fremitus similar to that transmitted to the ear applied to the phenendoscope placed against a trembling muscle. A slight pulsation not quite synchronous (retarded) with that of the facial artery could be felt. Aneurism of the inferior branch of the inferior cervical artery situated beneath the plate vein was diagnosed. If the arterial dilatation did not extend too high up toward the common trunk of this artery, it might have been possible to ligate the vessel and reduce the tumor.—*Rec. de Méd. Vét.*

